# Penile Scintigraphy—A Diagnostic Method for Vasculogenic Erectile Dysfunction

**DOI:** 10.3390/medsci13040208

**Published:** 2025-09-24

**Authors:** Nina Kulchenko, Daniil Yuferov, Farid Mangutov, Dmitri Kruglov, Elina Korovyakova, Petr Shegai, Andrei Kaprin, Grigory Demyashkin

**Affiliations:** 1 Department of Digital Oncomorphology, National Medical Research Centre of Radiology, 2nd Botkinsky Pass., 3, 125284 Moscow, Russia; kle-kni@mail.ru (N.K.); faridv7@gmail.com (F.M.); kruglov@yandex.ru (D.K.); dr.shegai@mail.ru (P.S.); kaprin@mail.ru (A.K.); 2 Research and Educational Resource Center for Immunophenotyping, Digital Spatial Profiling and Ultrastructural Analysis Innovative Technologies, Peoples’ Friendship University of Russia (RUDN University), Miklukho-Maklaya Str. 6, 117198 Moscow, Russia; elina.korovyakova@mail.ru; 3 Laboratory of Histology and Immunohistochemistry, Institute of Translational Medicine and Biotechnology, I.M. Sechenov First Moscow State Medical University (Sechenov University), Trubetskaya Str. 8/2, 119048 Moscow, Russia; yuferov.dy@dvfu.ru; 4 Department of Urology and Operative Nephrology, Peoples’ Friendship University of Russia (RUDN University), Miklukho-Maklaya Str. 6, 117198 Moscow, Russia

**Keywords:** penile scintigraphy, erectile disfunction, CDUS, SPECT, vasculogenic erectile disfunction, arterial insufficiency, veno-occlusive erectile disfunction, microcirculation disfunction, venous insufficiency

## Abstract

**Background:** Erectile dysfunction (ED) is a disease whose occurrence is steadily increasing worldwide. This pathology is multifactorial and often combined with other diseases. ED of organic genesis in 50–80% of men is vasculogenic. **Methods**: A survey was conducted of 88 men (aged 44 to 62) who complained of erectile dysfunction. It consisted of a questionnaire administered according to the protocols “International Index of Erectile Function” and “Aging Male Screening”, and was followed by a color Doppler ultrasound (Logiq 9 ExpertGE with a 7 MHz linear transducer using B mode) and penile scintigraphy (single-photon emission computed tomography). The procedures were initially performed at rest, then during pharmacologically induced erection, which was achieved through the intake of phosphodiesterase-5 (PDE5) inhibitors. Patients who did not respond to pharmacological stimulation and had IIEF scores below 5–7 were offered surgical treatment—penile prosthesis followed by histological examination of the tissue of the corpus cavernosum. Statistical analysis was carried out using Microsoft Excel and STATISTICA 10.0 software. The Mann–Whitney U test was used to assess differences between quantitative variables, with the significance level set at *p* ≤ 0.05. **Results**: Penile scintigraphy shows high sensitivity (85.2%) and specificity (83.3%), outperforming color Doppler ultrasonography in detecting vasculogenic ED. **Conclusion**: Penile scintigraphy is demonstrated to be a highly informative method, allowing us to analyze the condition of the magistral and organ blood flow, as well as the microcirculatory bed of the cavernous bodies of the penis. This improves the effectiveness of this method in diagnosing various types of vasculogenic erectile dysfunction (ED), which opens opportunities for its use together with ultrasound examination when the latter is less informative.

## 1. Introduction

Erectile dysfunction (ED) is a disease whose occurrence is steadily increasing worldwide. This pathology is multifactorial and often combined with other diseases, among which the most important are chronic diseases of the cardiovascular, endocrine and urinary systems; age; and psychological problems. ED of organic genesis in 50–80% of men is vasculogenic [1,2]. Given the scale of the problem and the sensitivity of the situation, ED diagnosis requires a multifaceted approach.

Examination of patients with this pathology includes questionnaires (such as the International Index of Erectile Function), as well as laboratory and instrumental studies [3,4]. Today, there are many methods that are available, and each of them has its own disadvantages and advantages.

For most urologists, Doppler ultrasound against the background of a pharmacological test (prostaglandin E1), as well as computed tomography (CT) or magnetic resonance imaging (MRI) of the penis in combination with intracavernous angiography, remains one of the leading methods of ED diagnosis [5].

Since ED is a sensitive issue for many men, minimally invasive methods are prioritized for investigating erectile function disorders. Among these diagnostic methods, penile Doppler ultrasound (PDU) is undoubtedly the most used. Despite its wide availability and low cost, penile Doppler ultrasound has several drawbacks: the lack of standardized protocols for its execution, the risk of priapism following intracavernous injection of prostaglandin E1, injection-related pain, and a high dependence of the accuracy of the examination on the physician’s expertise and the quality of the ultrasound equipment. All of this leads to significant variability in the data among different urological institutes [6,7].

The main disadvantages of most methods of ED diagnosis with contrast are invasiveness and psycho-emotional stress in the patient, and pain experienced when carrying out this manipulation. Any surgical manipulation, even if minimally invasive, is accompanied by the risk of complications such as bleeding, hemorrhage, soft-tissue hematoma, and the development of fibromyalgia of the penis in the future [8,9].

Considering the above, the search for instrumental methods to investigate erectile dysfunction, especially the most common type—vasculogenic ED—is important. These methods should be accessible, informative and safe, and possess high accuracy and specificity.

Among imaging techniques used to diagnose erectile dysfunction (ED), scintigraphy stands out alongside CT and MRI. As a radionuclide imaging method, scintigraphy has a few unique advantages for evaluating regional blood flow (perfusion) across various organs and tissues compared to other imaging modalities. Scintigraphy enables us to assess the blood supply to an organ at the level of the microcirculatory bed, whereas other methods give mainly anatomical information. This technique allows us to make an accurate assessment of parameters such as perfusion intensity (the volume of blood delivered per unit time per unit tissue volume), blood flow distribution within different regions of the organ, and venous outflow velocity. Penile scintigraphy has been proven to have high sensitivity to changes in organ blood flow, detecting even minor perfusion disturbances that may not be evident as structural changes on CT or MRI [10,11]. Scintigraphy has proven valuable in diagnosing conditions related to organ vascularization, including myocardial infarction [12] and kidney diseases [13]. In andrology, scintigraphy has been successfully applied to assess testicular blood flow in cases of testicular torsion [4].

Penile scintigraphy is a promising and optimal diagnostic method that can be used to visualize various forms of ED with minimal invasiveness. However, it also has its shortcomings, which create controversy when deciding on a diagnostic method, which will be discussed further later in this paper.

Thus, the lack of an answer to the question of which method is best for diagnosing vasculogenic ED contributes to the relevance of this topic today.

The aim of this study is to improve the results of erectile dysfunction diagnosis by using a minimally invasive method—penile scintigraphy.

## 2. Methods

### 2.1. Patients

The study sample comprised a group of 88 men aged 44 to 62 (mean age, 53 ± 9 years). All these patients complained of impaired erection quality. All patients were subjected to the International Index of Erectile Function (IIEF) and Aging Male Screening (AMS) protocols. In addition, the study included a group of healthy volunteers (n = 19).

*Inclusion criteria:* Patients with ED who had IIEF scores below 22.

*Exclusion criteria:* Patients whose AMS scores were greater than 26 and patients who suffered from hormonal, infectious, genetic, and oncological diseases that cause erectile dysfunction.

### 2.2. Design

Every patient (n = 88) underwent color Doppler ultrasound (CDUS) and penile scintigraphy. CDUS was performed by a Logiq 9 ExpertGE (GE Healthcare, Chicago, IL, USA) with a 7 MHz linear transducer using B mode. Penile scintigraphy was performed via ^99m^Tc single-photon emission computed tomography (SPECT).

### 2.3. Algorithm

Both CDUS and penile scintigraphy were performed according to a fixed algorithm. They were initially performed at rest, then during pharmacologically induced erection, which was achieved by taking a phosphodiesterase-5 (PDE-5) inhibitor (Sildenafil 100, Teva Pharmaceutical Industries, Petah Tikva, Israel).

### 2.4. Penile Scintigraphy

**Figure 1:**
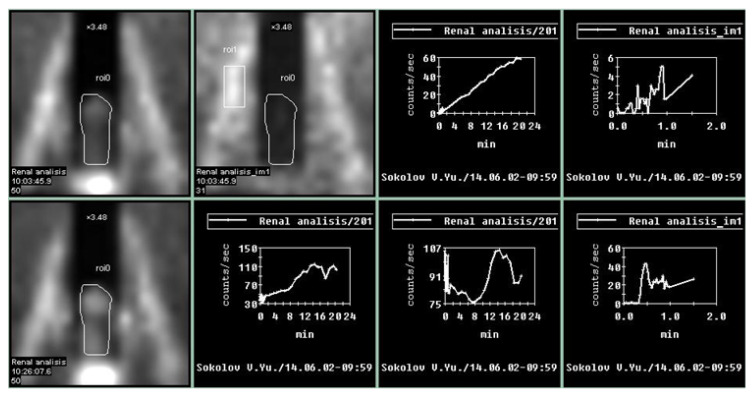
Normal parameters of the penile scintigraphy: arterio-organ transit time of the radiopharmaceutical drug is fast, at 3 s; the arterial and venous segments are clearly defined. An ascending segment is defined on the integral curve, with preserved phase oscillations without clearly expressed descending segments. On curves obtained after pharmacological stimulation, there is an increase in contrast accumulation activity up to 200%.

Penile scintigraphy includes dynamic recording of the progress of the indicator bolus and obtaining a series of static images (1–2 frames per second) (Figure 1). The study was performed using a single-photon emission computed tomography (SPECT) system Siemens Simbia E (Siemens Medical Solutions USA, Inc., Hoffman Estates, IL, USA). Scintigraphy of the penis was initially carried out at rest and subsequently during pharmacologically induced erection. The upper boundary of the gamma camera field of view was positioned at the level of the umbilicus, while the lower boundary extended to the proximal third of the thigh. After a bolus injection of ^99m^Tc-pertechnetate into the cubital vein, a series of images was obtained for 1–2 min in a 64 × 64 matrix (with an acquisition rate of 1–2 frames per second). Beginning at 2 min, image acquisition continued at a rate of 1 frame per minute. The total duration of scintigraphic recording was 15–20 min. For quantitative assessment, time–activity curves were generated over the penile region with background subtraction and decay correction. These included an integral penile time–activity curve (histogram) at rest without pharmacological stimulation, a vascular-phase fragment of the penile time–activity curve (0–2 min), and a vascular-phase control time–activity curve obtained over the iliac artery bifurcation.

Upon erection, the second phase of the study was performed: penile scintigraphy under pharmacological stimulation. The protocol included dynamic scintigraphy followed by acquisition of a series of static images over 20 min, in a mode analogous to that of the resting study. The total duration of scintigraphic recording ranged from 20 to 60 min. For evaluation, a penile time–activity curve was generated with background subtraction and decay correction.

An integral penile time–activity curve (histogram) was obtained starting from the onset of erection following pharmacological stimulation.

According to dosimetry data, the dose absorbed by the ovaries during standard testing (150–300 MBq) ranges from 2 to 3 mGy, which is significantly lower than the dose received in routine pyelography (4–8 mGy). This demonstrates the safety of penile scintigraphy in ED diagnosis.

Patients who did not respond to pharmacological stimulation and had IIEF scores below 5–7 (severe erectile disfunction) were offered surgical treatment—penile prosthesis.

Statistical analysis was carried out using Microsoft Excel and STATISTICA 10.0 software. The Mann–Whitney U test was used to assess differences between quantitative variables, with the significance level set at *p* ≤ 0.05.

To evaluate the diagnostic performance of the two diagnostic methods (ultrasound and penile scintigraphy), standard metrics were applied: sensitivity, specificity, and accuracy. Sensitivity (%) was defined as the proportion of true positive results relative to the total number of patients. Specificity (%) was defined as the proportion of true negative results among patients without the condition. Accuracy (%) was defined as the proportion of true positive and true negative results relative to the total number of patients with and without the condition.

### 2.5. Ethical Statement

The Local Ethics Committee of RUDN University approved this study (Protocol №7 from 22 September 2016). All samples and data were handled in strict accordance with the Guidelines on Human Biobanks and Genetic Research Databases, ensuring proper collection, anonymization, processing, and storage under internationally recognized ethical and quality standards. This article adhered to the Strengthening the Reporting of Observational Studies in Epidemiology (STROBE) guidelines. All procedures followed the principles of the Declaration of Helsinki.

## 3. Results

### 3.1. Interview and Questionnaires

All examined patients (n = 88) reported a decrease in the quality and frequency of spontaneous erections, as well as delayed penile tumescence during sexual intercourse (Table 1).

The analysis of the IIEF questionnaire results showed that 24 (27.3%) patients had mild ED (nearly 1 in 4 surveyed men), 28 (31.8%) patients had mild to moderate ED, 21 (23.9%) males had moderate ED, and 15 (17.0%) patients had severe ED (χ^2^ = 5.40, *p* = 0.021). Thus, the chances of detecting erectile dysfunction using the IIEF questionnaire are 3.21 (OR = 3.00; 95% confidence interval [CI]: 1.16 to 7.70). However, the IIEF questionnaire does not allow for determination of the specific type of ED.

### 3.2. Color Doppler Ultrasound (CDUS)

**Figure 2:**
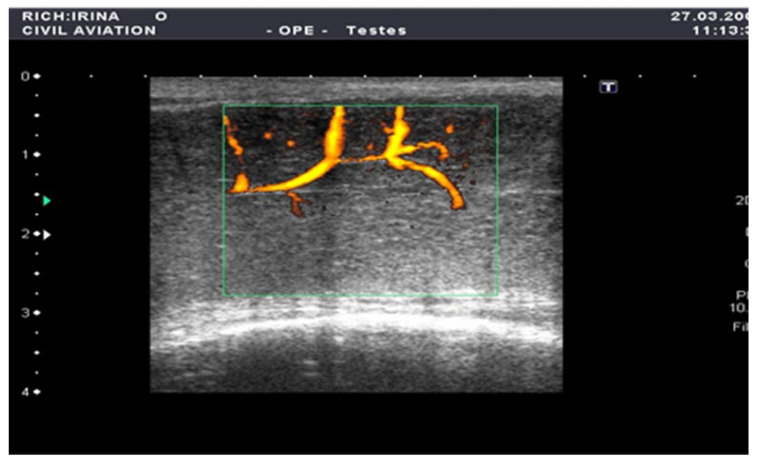
Penile CDUS during pharmacologically induced erection. Signs of arterial insufficiency: visualization of arterial stenosis areas, with spiral arteries showing uneven and asymmetric contrast enhancement.

**Figure 3:**
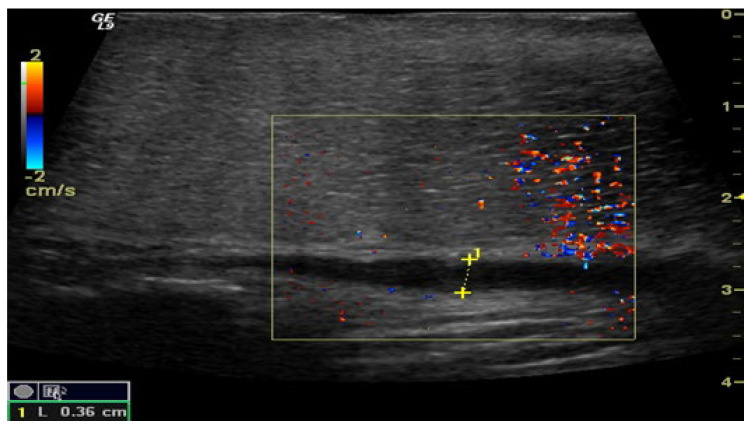
Penile CDUS during pharmacologically induced erection. Signs of veno-occlusive ED: diameter of the deep dorsal vein is enlarged, indicating venous insufficiency.

The penile CDUS revealed that 50 (56.8%) patients had ED signs, and 38 (43.2%) patients had no structural changes. Among these fifty males, 22 (25%) patients had penile arterial insufficiency (Figure 2), 17 (19.3%) patients had veno-occlusive ED (Figure 3), and 11 (12.5%) men had microcirculation disorder in the cavernous bodies of the penis (χ^2^ = 10.68, *p* = 0.002). Thus, the odds of detecting ED by using pharmacologically stimulated CDUS are 2.00 (OR = 4.47; 95% CI: 1.77 to 11.24).

The correct blood flow velocity measurements (Table 2), which determine the subtype of vasculogenic pathology of the penis, can be obtained only with pharmacologically induced erection.

The results of penile CDUS showed that this type of study has low sensitivity (54.5%) and specificity (60.5%) (Table 3). At the same time, penile CDUS allows us to identify the type of vasculogenic ED, with the highest chance of visualizing disorders of arterial blood flow in the penis (Table 4).

### 3.3. Penile Scintigraphy

The penile scintigraphy revealed that 12 (13.6%) patients had no structural changes (χ^2^ = 6.09, *p* = 0.014). The following features were observed: a distinct arterial segment was visualized on the curve of the external iliac artery; the arterio-organ transit time was 3.4 ± 0.9 s (within normal limits); arterial and venous segments were clearly delineated; the integral curve exhibited a pronounced ascending phase with preserved phase oscillations and lacked clearly defined descending phases; and curves obtained 20–60 min post-stimulation showed an increase in radiopharmaceutical uptake activity by more than twofold within 20–32 min (Figure 1).

**Figure 4:**
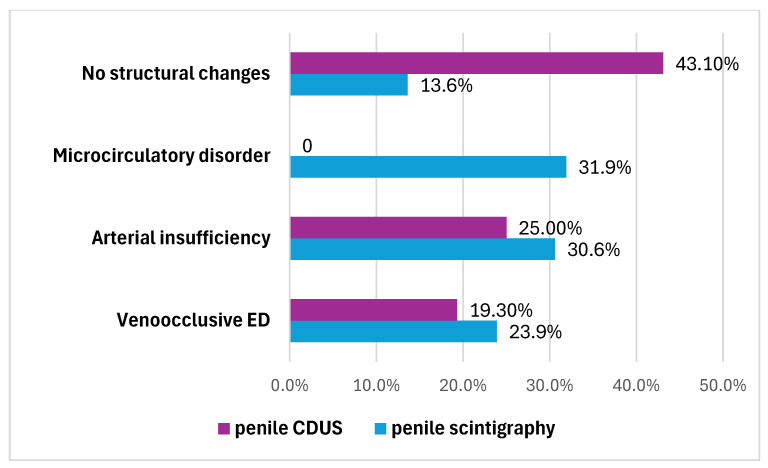
Types of ED that have been identified by penile scintigraphy and penile CDUS.

Another 76 (86.4%) patients had ED signs according to penile scintigraphy, the distribution of which is also illustrated in Figure 4. They were characterized as follows:

- Twenty-seven (30.6%) patients had penile arterial insufficiency—manifesting as a slowdown in transit time (up to 13.6 ± 2.7 s; normal range: 3–7 s), smoothing of the ascending segment and displacement of the arterial peak by the end of the first minute.
- Twenty-one (23.9%) patients had veno-occlusive ED—manifesting as an acceleration in transit time (up to 1.8 ± 1.4 s), a smoothly ascending segment and a lack of characteristic phase venous oscillations, with the presence of an elongated descending segment (4.3 ± 1.5 s).
- Twenty-eight (31.9%) men had microcirculation disorder in the cavernous bodies of the penis—manifesting as the absence of typical phase oscillations on the integral curve and a pronounced ascending segment with smoothed phase oscillations and no clearly defined descending segment.

Thus, the odds of detecting erectile dysfunction (ED) using penile scintigraphy during pharmacologically induced erection are 2.25 (OR = 3.00; 95% CI: 1.23 to 7.26).

**Figure 5:**
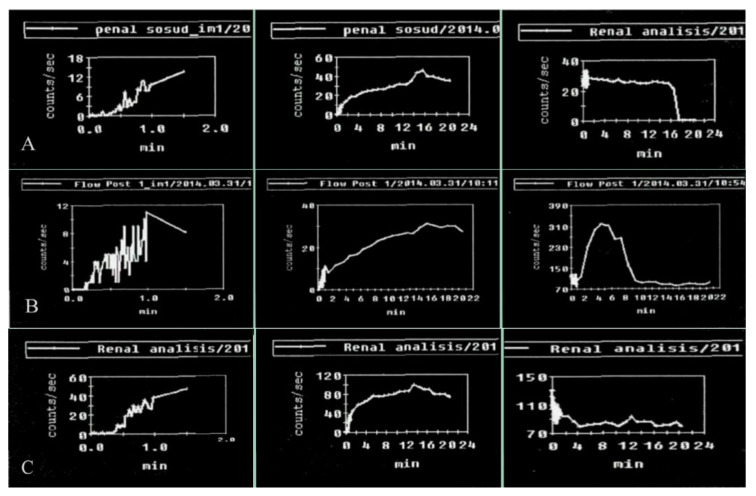
Results of penile scintigraphy in the visualization of vasculogenic erectile dysfunction: (**A**) arterial insufficiency; (**B**) venous insufficiency; (**C**) microcirculation disorder.

Thus, during this study of a cohort of 88 men with ED using an instrumental method such as penile scintigraphy, a distribution of patients by type of vasculogenic ED was obtained, as shown in Figure 5.

During the study, the transit time of the radiotracer for different types of ED was also determined (Table 5).

### 3.4. Findings

Penile scintigraphy shows high sensitivity (85.2%) and specificity (83.3%) in the diagnosis of ED (Table 6). Scintigraphy allows us to identify the type of vasculogenic ED, with the highest chance of visualizing disorders of microcirculation and arterial blood flow in the penis (Table 7).

Based on the conducted research, the following advantages of penile scintigraphy can be highlighted: it is minimally invasive, allows a bolus injection of the contrast agent into the antecubital vein, enables a holistic view of the main and organ blood flow, may allow the assessment of arterio-organ transit, involves low radiation exposure and reduces subjectivity in interpreting the study results, thereby reducing the risk of medical errors

Patients (n = 4; 4.5%) who continued to complain of erectile dysfunction and who did not respond to pharmacological stimulation were recommended surgical treatment with penile prosthesis.

## 4. Discussion

This study is devoted to the evaluation of the effectiveness of penile scintigraphy as a method for diagnosing vasculogenic ED.

Most authors consider penile CDUS to be the universal method for the evaluation of ED. However, penile CDUS does not allow simultaneous assessment of blood flow velocity throughout the entire organ, as measurements are performed at localized points corresponding to individual vessels’ anatomical projections.

Other diagnostic methods for vasculogenic ED, such as cavernosography, computed tomography and magnetic resonance imaging, are often recommended by authors as second-line diagnostic tools following ultrasonography. However, these techniques typically require the administration of contrast agents, often via intracavernous injection, to enhance vascular visualization. This diagnostic approach is highly invasive and frequently associated with complications, including bleeding, soft tissue hematomas of the penis, and focal fibrosis of the corpora cavernosa. Therefore, in modern instrumental diagnostics of ED, it is important not only to visualize penile blood flow but also to seek effective diagnostic methods that ensure maximal safety in contrast agent administration.

All patients in the study group (n = 88) were submitted to a penile CDUS, and 50 (56.8%) of them showed signs of ED, 22 (25%) had arterial insufficiency, 17 (19.3%) had veno-occlusive ED, 11 (12.5%) had mixed ED, and 38 (43.2%) had no pathological structural changes.

To further investigate the causes of penile blood insufficiency, all patients underwent radioisotope examination—penile scintigraphy.

After penile scintigraphy, other results were obtained: 76 patients (86.3%) showed signs of ED, which exceeded the sensitivity of the ultrasound results by 30.7%. ED due to arterial insufficiency was detected in 27 patients (30.6%), veno-occlusive ED was detected in 21 patients (23.9%), microcirculation disorder in the cavernous bodies of the penis were detected in 28 patients (31.9%), and only 12 patients (13.6%) did not show any structural pathologies, which can be interpreted as neurogenic ED. Thus, penile scintigraphy detected arterial and venous flow disorders 3.8 times more often than color Doppler ultrasonography.

Similar results were obtained by us in pilot projects, as well as by other researchers on a smaller sample of patients, where the effectiveness of penile scintigraphy was also demonstrated. In addition, Chumakov P. (2019) notes that the use of the penile scintigraphy method helps reduce the risk of intracavernous complications and eliminates psychological stress in patients [9].

Thus, based on the conducted comprehensive study, penile scintigraphy is demonstrated to generally be a minimally invasive, highly effective and highly specific method of diagnosing various types of ED, surpassing the specificity of ultrasound by 22.8%. Among the minor (but easily solved) disadvantages of penile scintigraphy, it is necessary to note the complexity of implementation and the need for equipment from urological institutes.

**Figure 6:**
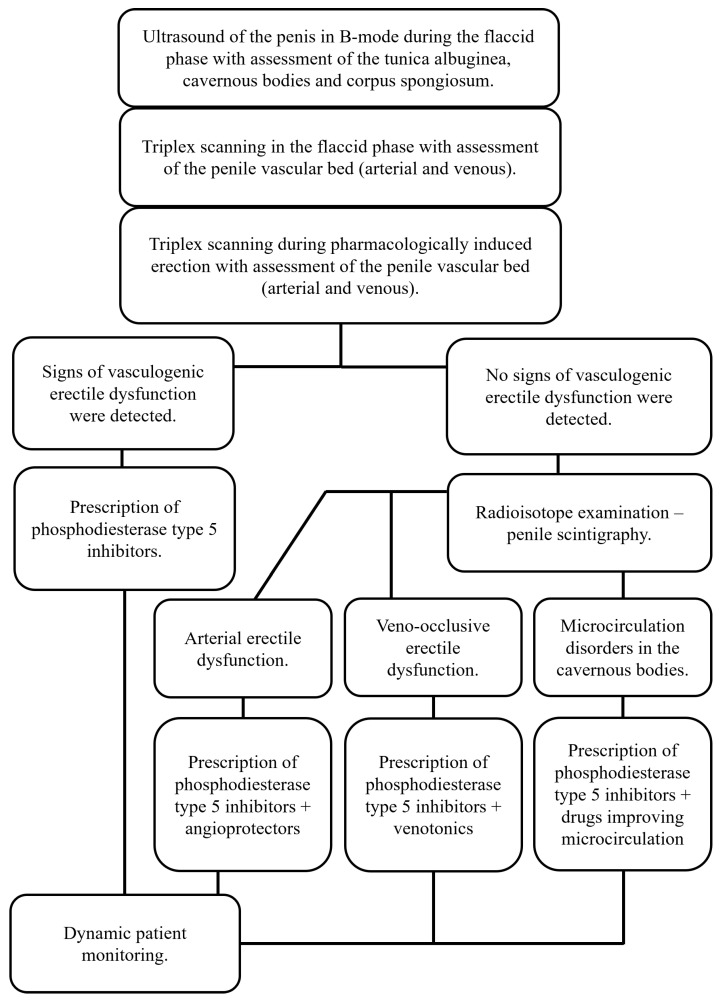
Algorithm for diagnosing vasculogenic erectile dysfunction.

Considering the lack of generally accepted standards for examining patients with erectile dysfunction, the analysis of the obtained results of vasculogenic erectile diagnostics using penile scintigraphy allowed us to propose a diagnostic algorithm for this category of patients (Figure 6), since it was proven to be effective in almost 87% of cases.

In conclusion, it should be noted that the study we conducted is the first in the world on this topic and has no analogs, and it demonstrates that penile scintigraphy is a promising method in urological practice.

## 5. Conclusions

Penile scintigraphy is demonstrated to be a highly informative method, allowing analysis of the condition of the magistral and organ blood flow, and the microcirculatory bed of the cavernous bodies of the penis. This improves its sensitivity in the diagnosis of various types of vasculogenic erectile dysfunction (ED), which opens opportunities for its use together with ultrasound examination when the latter is less informative.

The application of the developed algorithm for diagnosing vasculogenic erectile dysfunction with the complex use of ultrasound examination and penile scintigraphy allows the establishment of an accurate diagnosis in 86.4% of cases.

## Figures and Tables

**Table 1:** Main complaints of the patients (n = 88).

Complaints	Number of Patients (n = 88)	(%)
Weakening of spontaneous erections	43	48.8
Absence of spontaneous erections	7	7.9
Increased intervals between coitus	15	17.0
Detumescence before ejaculation	20	22.7
Increased duration of sexual stimulation required to achieve erection	47	53.4
Lack of self-confidence	41	46.6
Expectation of failure	52	59.0

**Table 2:** Ultrasound measurements of penis blood flow in a pharmacologically induced erection.

Arteria	Parameters	Patients with Vasculogenic ED (n = 88)	Healthy Volunteers (n = 19)
Cavernous artery	Peak blood flow velocity; Vmax (cm/s)	17 ± 2.1	34 ± 1.9
	Resistive index (RI)	0.75	0.8
	Pulsatility index (PI)	1.3 ± 0.09	3.8 ± 0.08
Dorsal artery	Peak blood flow velocity; Vmax (cm/s)	21 ± 1.2	36 ± 1.4
	Resistive index (RI)	0.76	0.82
	Pulsatility index (PI)	1.4	3.2 ± 0.28
Deep dorsal vein	Peak blood flow velocity; Vmax (cm/s)	9 ± 2.7	-
	End diastolic blood flow velocity; Ved (cm/s)	10 ± 3.7	-
	Resistive index (RI)	0.6 ± 0.12	-
	Pulsatility index (PI)	2.7 ± 0.11	-

**Table 3:** Sensitivity and specificity of penile CDUS in the visualization of erectile dysfunction.

Signs	(n)
True Positive Result	48
False Positive Result	15
True Negative Result	23
False Negative Result	2
Total Patients	88

In these results sensitivity of penile CDUS is 54.5%, and specificity of penile CDUS is 60.5%.

**Table 4:** The chances of detecting a specific type of vasculogenic erectile dysfunction using penile CDUS.

Type of ED	Chance of Detecting ED	Odds Ratio (OR)	Standard Error (SE)	CI 95%	
				Min	Max
Arterial insufficiency	1.57	0.59	0.46	0.23	143
Venous insufficiency	1.06	0.47	0.45	0.19	1.15
Microcirculation disorder	0.57	0.30	0.46	0.12	0.76

**Table 5:** Transit time of radiotracers in patients with vasculogenic ED (*p* < 0.05).

Subtypes of Vasculogenic ED	Patients		Radiopharmaceutical Evacuation (s)
	n	%	
Arterial insufficiency (s)	27	30.6	13.6 ± 4.0
Veno-occlusive ED (s)	21	23.6	4.3 ± 1.5
Microcirculation disorder in the cavernous bodies of the penis (s)	28	31.9	7.5 ± 3.6
No structural changes (s)	12	13.6	3.4 ± 0.9

**Table 6:** Sensitivity and specificity of penile scintigraphy in the visualization of erectile dysfunction.

Signs	Patients (n)
True Positive Result	75
False Positive Result	2
True Negative Result	10
False Negative Result	1
Total (patients)	88

In these results, sensitivity of penile scintigraphy is 85.2%, and specificity of penile scintigraphy is 83.3%.

**Table 7:** The chances of detecting a specific type of vasculogenic erectile dysfunction using penile scintigraphy.

Type of ED	Chance of Detecting ED	Odds Ratio (OR)	Standard Error (SE)	CI 95%	
				Min	Max
Arterial insufficiency	1.12	2.34	0.44	0.91	5.65
Venous insufficiency	0.77	1.81	0.45	0.74	4.39
Microcirculation disorder	1.16	2.33	0.45	0.96	5.63

## Data Availability

The original contributions presented in this study are included in the article. Further inquiries can be directed to the corresponding author.
